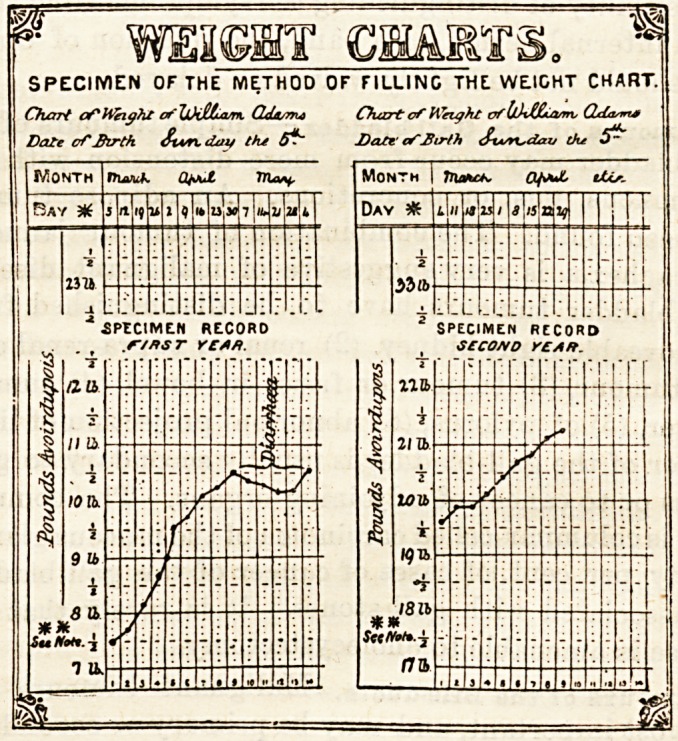# New Appliances and Things Medical

**Published:** 1897-10-30

**Authors:** 


### NEW APPLIANCES AND THINGS MEDICAL.

[We shall be glad to receive, at our Office, 28 & 29, Southampton Street, Strand, London, W.C., from the manufacturers, specimens of all new preparations and appliances which may be brought out from time to time.]

### HARPER'S PATENT CHAMBER RIM CORK CUSHION.

(The Hygienic Appliances Company, 58, Renfield Street, Glasgow.)

This new invention is intended to be fitted to the rim of the chamber utensil, and, being made of a non-conducting material, to thus obviate chill or shock through contact with cold earthenware. The cork is antiseptically treated, and is intended to be kept so by the use of a sanitary powder which is supplied—if soiled it can be scrubbed without injury. It does not particularly appeal to us as a practical or cleanly arrangement; with careless servants or nurses it might introduce sources of danger.

### URINARY TEST CASE.

(Parke, Davis, and Co., 21, North Audley Street, Grosvenor Square, W., and Detroit, Mich.)

In a very handy form, so that it can easily be carried in the pocket, Messrs. Parke, Davis, and Co. have recently devised a urinary test case. It contains all necessary reagents in the form of tablets, and a new form of urinometer which is sufficiently accurate for clinical purposes, and which consists of a chain of specific gravity beads contained in a small tube with a hole at the bottom, so that all that is necessary for making an estimation is to dip the tube into a second and larger one containing the urine. The specific gravity of the specimen is read off by observing which of the beads float and which of them sink—for instance, if three beads float we know that the specific gravity of the fluid is between 1·015 and 1·020. The tests which can be applied by the reagents contained in the case are: three for sugar, namely—(1) Indigo carmine, (2) picric acid, (3) bismuth subnitrate; and four for albumin—(1) Potassio mercuric iodide, (2) sodium tungstate, (3) potassium ferrocyanide, (4) picric acid. In addition to the foregoing a quantitative estimation of albumin can also be arrived at by the method of Dr. Oliver; the colour of the urine can be noted according to Dr. Vogel's scale of tints; and the reaction of the urine discovered by the use of litmus paper. That a veritable chemical laboratory can be compressed into so small a space is a remarkable tribute to latter-day ingenuity and to the enterprise and skill of Messrs. Parke, Davis, and Co. It is needless to indicate the value of such a reagent case to the busy country practitioner.

### TWO NEW CLINICAL CHARTS.

(Messrs. Reynolds and Branson, 13, Briggate, Leeds.)

The first of these, which may be called the "Medical Recording Calendar," is practically a calendar chart with the days, weeks, and months of a whole year condensed within small compass, with marginal space for notes. In the treatment of any long-continued illness, say of epilepsy, malaria, or irregular menstrual trouble, it is of the greatest assistance to the physician to have a tabular record of the disturbances in order that at a glance he may be able to follow the course of the disease. For its use all that is necessary is that the patient or nurse shall make a cross or other explanatory mark in the space corresponding to the day on which the phenomenon occurs. The history of a year may in this way be condensed into one page. The second chart, and one equally useful, is for the weekly record of an infant's weight from the day it is born until the end of the first year. The value of such a "weight curve" is one that can hardly be overestimated, showing, as it does in graphic form, the advantages or the reverse of any particular kind of food, climate, or other hygienic surrounding. An infant's weight is peculiarly sensitive to slight modifications in external surroundings, and the study of such charts will be particularly instructive to those who have the care of children.

## Figures and Tables

**Figure f1:**
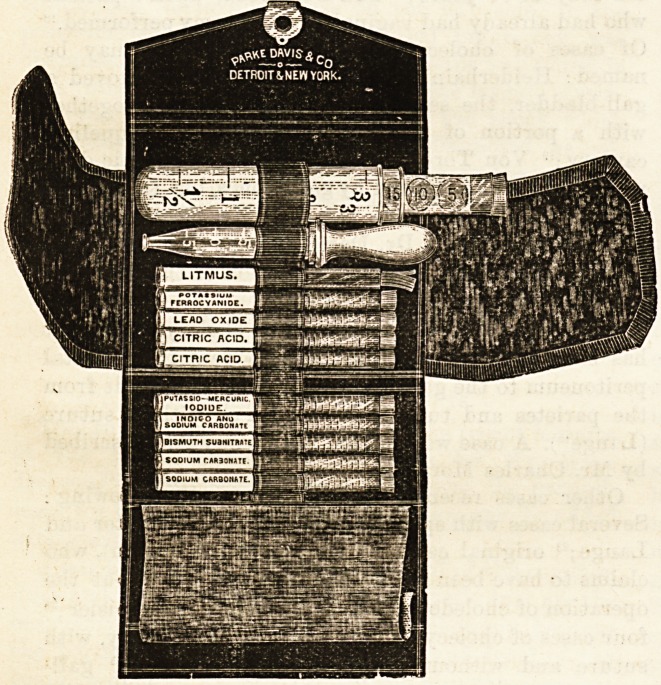


**Figure f2:**
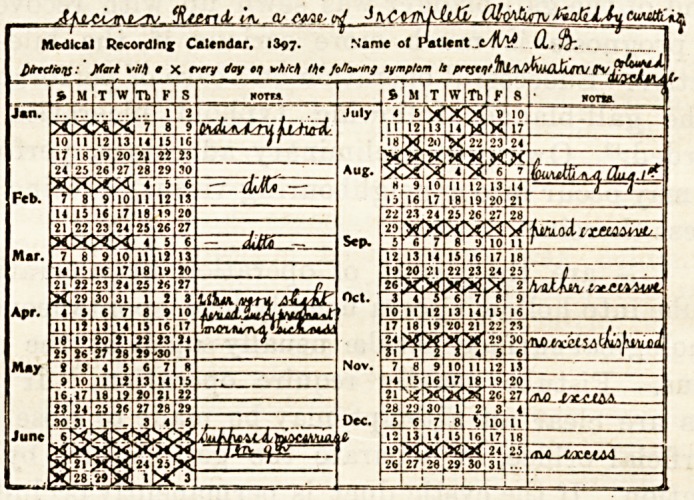


**Figure f3:**